# Association Between Blood Culture Bottle Shortage and Ordering Restrictions and Clinical Outcomes for Patients With *Staphylococcus aureus* Bacteremia

**DOI:** 10.1093/ofid/ofaf546

**Published:** 2025-09-15

**Authors:** Romney M Humphries, Ritu Banerjee, William D Dupont, David Gaston, Nicholas McKenzie, Michael Petit, W Dale Plummer, Matthew W Semler, Caroline Taylor, Thomas R Talbot

**Affiliations:** Department of Pathology, Microbiology and Immunology, Vanderbilt University Medical Center, Nashville, Tennessee, USA; Division of Pediatric Infectious Diseases, Department of Pediatrics, Vanderbilt University Medical Center, Nashville, Tennessee, USA; Department of Biostatistics, Vanderbilt University Medical Center, Nashville, Tennessee, USA; Department of Pathology, Microbiology and Immunology, Vanderbilt University Medical Center, Nashville, Tennessee, USA; Division of Infectious Diseases, Department of Medicine, Vanderbilt University Medical Center, Nashville, Tennessee, USA; Department of Pathology, Microbiology and Immunology, Vanderbilt University Medical Center, Nashville, Tennessee, USA; Department of Pathology, Microbiology and Immunology, Vanderbilt University Medical Center, Nashville, Tennessee, USA; Department of Biostatistics, Vanderbilt University Medical Center, Nashville, Tennessee, USA; Division of Allergy, Pulmonary, and Critical Care Medicine, Vanderbilt University Medical Center, Nashville, Tennessee, USA; Center for Learning Healthcare, Vanderbilt Institute for Clinical and Translational Research, Nashville, Tennessee, USA; Department of Enterprise Analytics, Vanderbilt University Medical Center, Nashville, Tennessee, USA; Division of Infectious Diseases, Department of Medicine, Vanderbilt University Medical Center, Nashville, Tennessee, USA

**Keywords:** bacteremia, blood cultures, sepsis, *Staphylococcus aureus*

## Abstract

**Background:**

A nationwide shortage of blood culture bottles led to significant restriction of blood culture utilization at our institution.

**Methods:**

We evaluated the impact of 3 combined interventions: (1) guidance on appropriate blood culture utilization, (2) restriction of repeat cultures within a 48-hour period, and (3) restriction of initial assessment to a single blood culture set consisting of 1 aerobic and 1 anaerobic blood culture bottle, on the management of patients with *Staphylococcus aureus* bacteremia (SAB) using an interrupted time series analysis.

**Results:**

Prior to the intervention, 90.1% of patients had 2 blood culture sets ordered for initial assessment, versus 5.7% during the intervention and 84.1% postintervention. The median number of cultures to document SAB clearance was 4 (range, 2–17) in the preintervention period, 2 (range, 2–9) during the intervention period, and 4 (range, 3–11) postintervention. The median number of days to SAB clearance was not significantly different across the study periods, nor were days to central venous catheter placement or days of intravenous *S aureus* therapy. Fewer patients had documented SAB clearance within 24 hours and median time to diagnosis for community-acquired cases was longer in the intervention period, suggesting the restrictions were not without potential clinical impact.

**Conclusions:**

These data demonstrate that efforts to reduce blood culture utilization should be implemented with careful stewardship in order to minimize adverse effects for patients with SAB.

## Teaser

Blood culture restrictions led to reduced culture use for patients with *Staphylococcus aureus* bacteremia and did not result in delays in documenting clearance or placement of central venous catheters or increased days of intravenous *S aureus* therapy.

*Staphylococcus aureus* is a leading cause of community- and hospital-onset bloodstream infections [1]. *Staphylococcus aureus* bacteremia (SAB) is life-threatening, with mortality rates up to 40% [2, 3]. Given the high rates of mortality and morbidity associated with SAB, standardized management is crucial for optimal patient outcomes and has been shown to reduce mortality up to 50% [4, 5]. Appropriate collection of blood cultures is a central component of the SAB management pathway. Yield of blood cultures for *S aureus* is optimal when at least 2 sets (with a set defined as 1 aerobic and 1 anaerobic bottle) are collected before initiation of antibiotics. Three sets are optimal for diagnosis of endovascular infection [6]. Blood cultures are often ordered 24–72 hours after initiation of antibiotic therapy to evaluate for treatment success, as SAB may persist beyond resolution of fever and other clinical signs of infection [7]. Repeatedly positive cultures are a predictor for metastatic infection [8], and are 1 sign used to define SAB as complicated. Uncomplicated SAB, in contrast, clears promptly following start of therapy. Duration of treatment is usually calculated from date of first negative blood culture, and placement of central venous catheters (CVCs) for intravenous (IV) antibiotic therapy is generally delayed until blood cultures are negative, due to the propensity of *S aureus* to colonize these devices.

In June 2024, Becton Dickinson announced a nationwide shortage of BACTEC blood culture bottles, creating an urgent need to restrict blood cultures to preserve availability of bottles to the highest-need cases. At our institution, a multidisciplinary team implemented 3 interventions to address the shortage [9]: (1) addition of health system–wide guidance on blood culture best practices to electronic order systems, which included guidance on scenarios where blood cultures are of low yield [10]; (2) restriction of repeat blood cultures within 48 hours; and (3) limiting the initial culture orders to a single set, as opposed to the standard 2–3 sets. We transitioned to an alternative blood culture vendor 6 weeks after the shortage commenced and lifted restrictions 8 weeks after they were first put in place. Throughout the shortage, every effort was made to mitigate patient risk, including exceptions to the ordering restrictions upon clinician request. Nonetheless, the restrictions used during the blood culture bottle shortage significantly reduced blood culture utilization and were outside the standards of care presented in national treatment guidelines [6, 11, 12]. We thus sought to evaluate the impact of these interventions on patient management and outcomes for adult patients with SAB.

## METHODS

### Study Setting

Vanderbilt University Medical Center (VUMC) includes 1139 licensed adult hospital beds across a large academic teaching hospital, 3 regional community hospitals, and a large regional network of ambulatory clinics. VUMC has active adult solid organ and stem cell transplant programs. Outside the shortage period, an average of 6000 blood culture sets are ordered per month in adult patients across VUMC, with 20%–25% of testing occurring in the adult emergency department (ED). All patients with SAB at the main teaching hospital had mandatory infectious diseases (ID) consultation. For the 3 regional hospitals, ID consultation was encouraged but not required.

### Study Design

Hospitalized adults (≥18 years of age) with *S aureus* recovered from blood cultures by our microbiology laboratory between 1 January 2024 and 31 October 2024 were evaluated in this study. An interrupted time series evaluation was used to assess the impact of the blood culture restrictions (intervention) on patient outcomes and management. Patients with *S aureus*–positive blood cultures in the 2 weeks following intervention start (1 July–14 July 2024) and in the 2 weeks after the intervention was ended (1 September–14 September 2024) were excluded, to provide a washout period for practice stabilization. Patients who received <48 hours of IV antibiotics with *S aureus* coverage were also excluded from analysis as these patients either died or left against medical advice within 48 hours of the blood culture collection, limiting data availability. In addition, patients with polymicrobial bacteremia were excluded. Pediatric patients were not included due to frequent use of single bottle (based on body weight) initial cultures.

Patient cohorts were divided into “preintervention” (1 January–30 June 2024), “intervention” (15 July–20 August 2024), and “postintervention” (15 September–31 October 2024) periods. In the preintervention period, there were no ordering restrictions on blood cultures. Blood culture order sets included options to order 1–3 blood culture sets (each set consisting of 1 aerobic PLUS and 1 anaerobic PLUS/F; BD, Sparks, Maryland, USA), with language to discourage ordering of single sets for initial diagnosis.

During the intervention, the following blood culture stewardship measures were put in place: (1) Provider education was added to the electronic medical record (EMR) order entry system, which nudged providers to consider the likelihood of bacteremia based on patient features [10, 13] (Supplementary Figures 1 and 2); (2) all blood culture orders were restricted to a single set; and (3) repeat blood cultures were prohibited if an order had been placed within the prior 48 hours. Adult emergency physicians were able to order 2 sets of blood cultures for patients who met VUMC-defined sepsis criteria at the main teaching hospital only. In addition, exceptions to the restrictions were available following consultation with the on-call clinical microbiologists; the option to consult with microbiology was presented to clinicians when they attempted to order repeat blood cultures within the 48-hour restriction period in the EMR.

In the postintervention period, we implemented a new vendor of blood cultures (VIRTUO, bioMérieux, Durham, North Carolina, USA) that was not experiencing a bottle shortage and order entry restrictions were removed from the EMR. Blood culture stewardship messaging remained in place. A new message alerted clinicians if a blood culture had been ordered within the past 48 hours, suggesting that repeat cultures may not be necessary, with exceptions listed, including SAB.

### Study Measures

All data were abstracted electronically from the EMR, and a random sample of 20% of cases was manually reviewed through chart review by a study team member. In addition, data were manually abstracted for source of bacteremia and assessment of complicated versus uncomplicated SAB, as defined elsewhere [14].

Outcomes of interest included time to first negative blood culture (ie, time to clearance of SAB), duration of IV anti–*S aureus* therapy, time to CVC placement, and number of blood cultures collected until first negative culture. Outcomes were calculated from time of the first blood culture collected on the hospital admission. Pitt bacteremia score was calculated as described elsewhere [15]. Additional outcomes evaluated included 30-day mortality, length of hospitalization and intensive care unit (ICU) hospitalization, incidence of recurrence of SAB >14 days after the incident blood culture [16], time from admission to diagnosis, and 30-day hospital-wide readmission data. Documentation of clearance was based on available blood culture draws. If no blood cultures were collected at 24 or 48 hours after the initial culture, this would be interpreted as not documented.

### Patient Consent Statement

This study was approved by the VUMC Institutional Review Board with waiver of consent as a quality improvement project.

### Statistical Analyses

Each outcome variable was assessed with linear models that regressed either the outcome, or the logarithm of the outcome, against time. Covariates were included in these models to permit separate slope and intercept parameters in the preintervention, intervention, and postintervention intervals. This permitted abrupt changes in the estimated response between the preintervention and intervention intervals and between the intervention and postintervention intervals. These models allowed separate rates of change with time of the response variable in these 3 intervals. Plots of the outcome, and the expected outcome, versus time showed that some of these response variables were skewed. Our choice of the response variable (linear or logarithmic) was driven by the model that gave the best fit to these models’ normality assumptions. When a logarithmic model was used, the observed and expected values were transformed back to the linear scale. The validity of extreme outliers was confirmed. Similar modes were also run restricted to patients with either complicated or uncomplicated SAB. Other measures were compared using 2-way analysis of variance.

## RESULTS

A total of 187 SAB events (184 unique patients) were evaluated, including 107 in the preintervention period, 36 during the intervention, and 44 in the postintervention period. Patients’ demographic information is presented in Table 1. During the intervention period, a smaller percentage of patients had complicated SAB (*P* = .53) and lower mean Pitt bacteremia scores (*P* = .06, Table 1) and lower incidence of MRSA (*P* = .09). All-cause 30-day mortality was numerically lower in the intervention period compared to the pre- and postintervention periods, but this was not statistically significant (Table 2). Total hospital days and ICU days did not differ across cohorts. Thirty-day all-cause readmission rates were higher in the intervention period (Table 2).

**Table 1.** Patient Demographic Data

Characteristic	Preintervention		Intervention		Postintervention		*P* value
	No.	%	No.	%	No.	%	
No.	107	…	36	…	44	…	
Age, y, median (range)	62	23.5	63.5	23.3	57	25.5	.45
Male sex, %	67.3	…	52.6	…	63.6	…	.28
Race							
White	87	81.3	28.0	77.8	34	77.3	.66
Black	11	10.3	6.0	16.7	7	15.9	
Hispanic/Latinx	5	4.7	1.0	2.8	2	4.5	
Other	4	3.7	1.0	2.8	1	2.3	
Patient location							.62
Main academic hospital	63	58.9	21	58.3	31	70.5	
Regional hospital	44	41.1	17	47.2	13	29.5	
ED	78	72.9	31	86.1	31	70.5	
ICU	9	8.4	1	2.8	2	4.5	
Source of bacteremia							.72
Endovascular	23	21.5	8	22.2	5	11.4	
CLABSI	10	9.3	6	16.7	8	18.2	
Pneumonia	6	5.6	0	0.0	0	0.0	
SSTI	21	19.6	3	8.3	10	22.7	
Abscess	7	6.5	2	5.6	3	6.8	
Osteomyelitis	13	12.1	7	19.4	4	9.1	
Device	9	8.4	4	11.1	4	9.1	
Other/unknown	18	16.8	8	22.2	10	22.7	
Complicated bacteremia	81	75.7	24	66.7	31	70.5	.53
Pitt bacteremia score							
Average	3.8	…	2.8	…	3.5	…	.06
0	6	5.6	1	2.8	3	6.8	
1	4	3.7	2	5.6	2	4.5	
2	26	24.3	12	33.3	9	20.5	
3	27	25.2	13	36.1	16	36.4	
≥4	44	41.1	8	22.2	14	31.8	
MRSA	50	46.7	11	30.6	24	55.5	.09

Abbreviations: CLABSI, central line–associated bloodstream infection; ED, emergency department; ICU, intensive care unit; MRSA, methicillin-resistant *Staphylococcus aureus*; SSTI, skin and soft tissue infection.

**Table 2.** Outcomes for Patients With *Staphylococcus aureus* Bacteremia in Relation to National Blood Culture Bottle Shortage

Characteristic	Preintervention		Intervention		Postintervention		*P* value
	No.	Median (IQR) or %	No.	Median (IQR) or %	No.	Median (IQR) or %	
Days to PICC placement	81	6.9 (5.7)	25	3.6 (1.4)	31	4.3 (3.9)	.76
Days of anti–*S aureus* IV therapy	107	28 (27)	36	30.5 (30)	44	28 (28)	.89
Days to first negative culture	99	2.8 (3.6)	35	2.5 (1.4)	39	2.7 (3.3)	.91
No. of culture sets ordered until first negative culture	99	4 (2.5)	35	2 (1)	39	4 (0.5)	<.05
Clearance within 48 h	37	37.4	9	25.7	13	33.3	.5
Clearance within 24 h	9	9.1	0	0	0	0	.02
No. of patients with 1 set for initial culture	10	9.3	33	94.3	7	15.9	<.05
Patients with repeat cultures ≤48 h from first culture	93	87.6	6	16.7	24	54.5	<.05
30-d all-cause mortality	18	16.8	2	5.6	5	11.4	.18
Length of hospitalization	14.9	11.6	12.3	7.0	13.5	11.0	.68
Duration of ICU stay	7.1	6.2	1.6	1.3	3.3	5.3	.45
Recurrence of SAB**^a^**	6	6.7	1	3.0	1	2.6	.69
30-d readmission**^a^**	19	21.3	10	30.3	8	20.5	.54

Abbreviations: ICU, intensive care unit; IQR, interquartile range; IV, intravenous; PICC, peripherally inserted central catheter; SAB, *Staphylococcus aureus* bacteremia.

^a^Recurrence rates and 30-day readmission rates were calculated using the number of patients who survived the incident admission.

**Figure 1.**
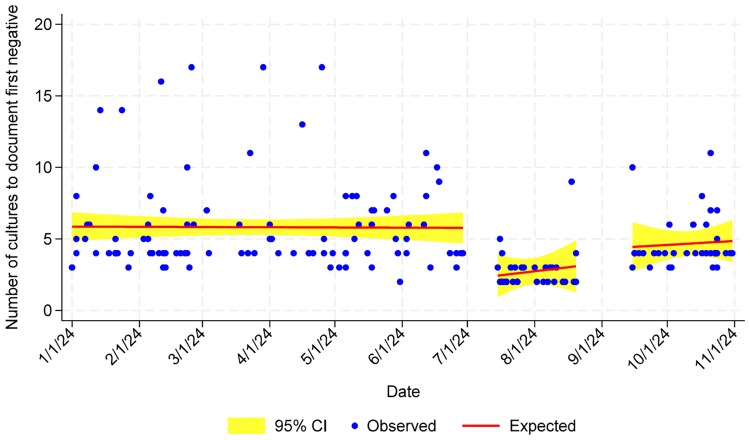
Number of cultures needed to document *Staphylococcus aureus* bacteremia clearance. Abbreviation: CI, confidence interval.

Prior to the intervention, 90.1% of patients had 2 blood culture sets ordered for initial assessment. In the shortage intervention period, this decreased to 5.7%. Postintervention, 84.1% had 2 blood culture sets ordered (Table 2). The median number of cultures to document SAB clearance, for those patients with clearance documented, was lower in the intervention period, but did not achieve statistical significance in linear regression analysis (Figure 1). Most patients had SAB diagnosed on the first blood culture set(s) collected in the ED. Excluding patients with hospital-onset infections (2 in the preintervention arm and 1 in the intervention arm), the median time to diagnostic culture collection from the first culture collected on admission was 0.96, 7.6, and 0.74 hours for patients in the preintervention, intervention, and postintervention periods, respectively. Evaluating only patients with delayed diagnosis (ie, those with a negative culture on initial admission), the median time to diagnostic culture collection from the first culture collected on admission was 10.7 hours (n = 3), 40.3 hours (n = 2), and 18.0 hours (n = 2), respectively (not shown). Each case with delayed diagnosis was manually reviewed. In the preintervention period, these cases were diagnosed on the third set of blood cultures, which were collected 2, 14, and 15 hours after the first 2 sets collected. In the intervention arm, 2 cases were diagnosed on a second set of cultures collected, 1 on a culture collected 26 hours after the first set by requesting an exception to the restrictions, and 1 collected 55 hours after the initial culture, once restrictions were lifted. Finally, both postintervention delayed cases were diagnosed on the third blood culture set, which was collected 3.8 hours and 32.2 hours after the initial set(s).

**Figure 2.**
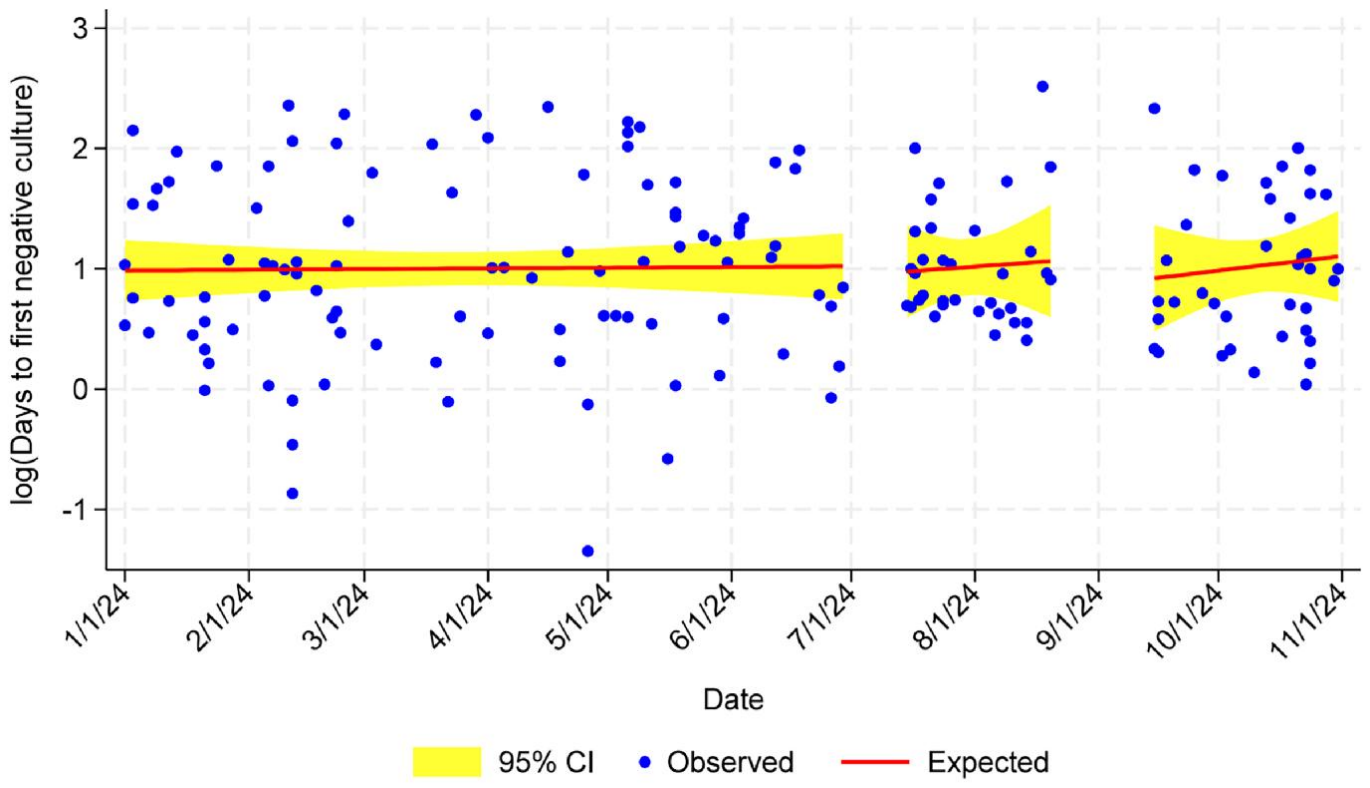
Days to first negative culture for patients with *Staphylococcus aureus* bacteremia. Abbreviation: CI, confidence interval.

The median number of days to SAB clearance was not significantly different across the study periods in linear analysis (Figure 2). We noted that no patients had SAB clearance documented within 24 hours of the index *S aureus*–positive blood culture in the intervention and postintervention periods. In contrast, 9 patients in the preintervention period (9.1%) had SAB clearance documented within 24 hours of the initial *S aureus*–positive culture (Figure 2, Table 2).

**Figure 3.**
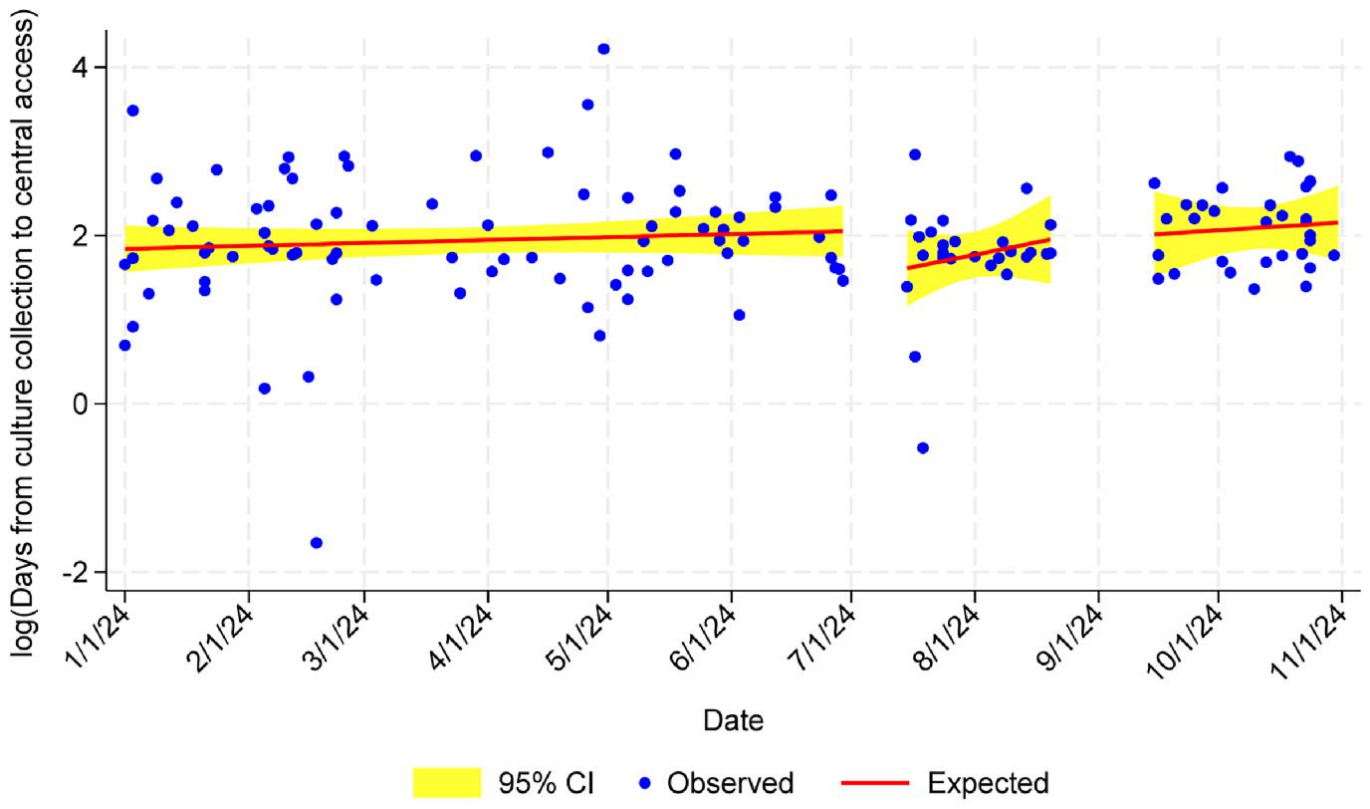
Days to central venous catheter placement. Abbreviation: CI, confidence interval.

**Figure 4.**
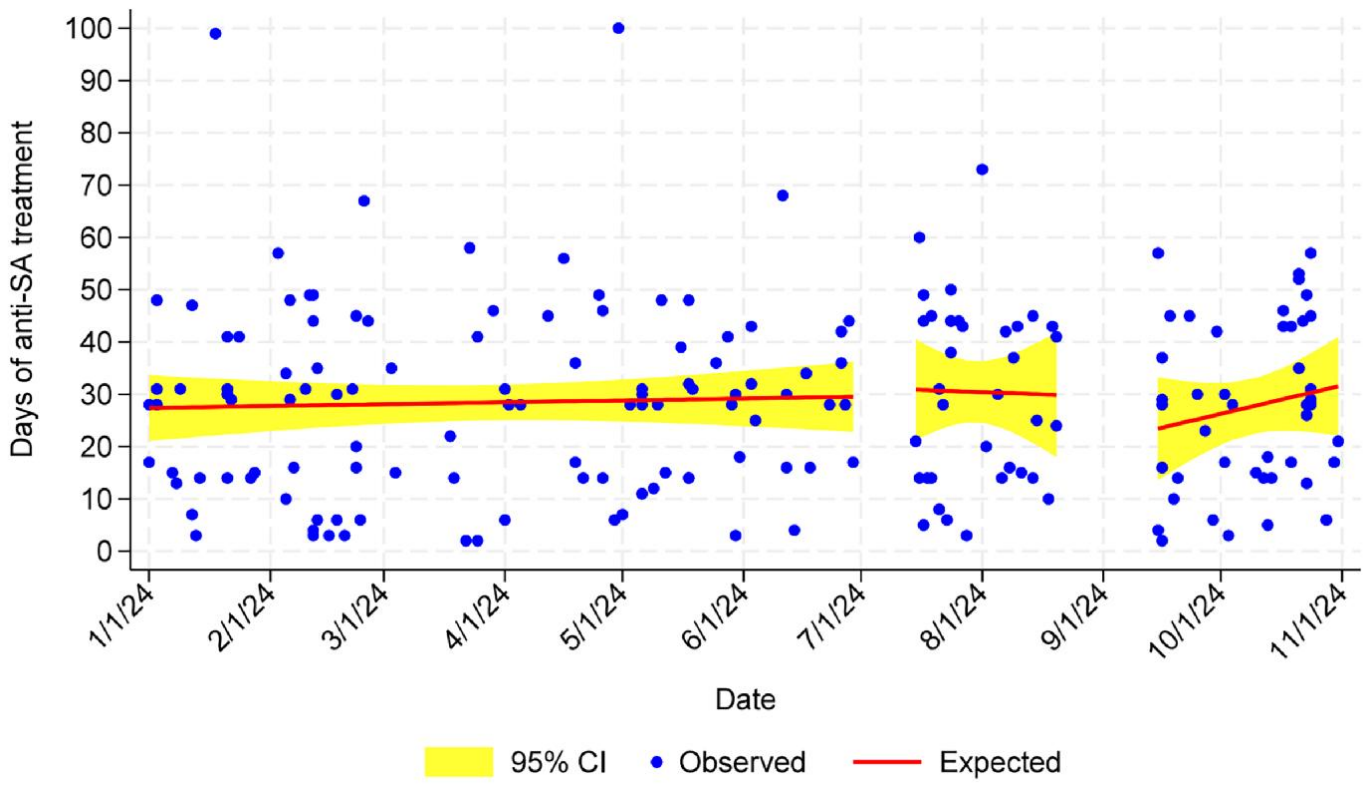
Days of anti-*Staphylococcus aureus* therapy. Abbreviations: CI, confidence interval; SA, *Staphylococcus aureus*.

Days to peripherally inserted central catheter placement was not significantly different between analysis periods (Figure 3, Table 2). Similarly, days of IV *S aureus* therapy did not differ across analysis periods (Figure 4, Table 2). None of these measures achieved statistical significance when patients with complicated and uncomplicated SAB were evaluated independently (not shown), nor when patients with Pitt bacteremia score ≥4 were evaluated.

## DISCUSSION

In this study, we evaluated the association between blood culture ordering restrictions that were implemented in response to a blood culture media shortage and the management and outcomes of patients with SAB. We found no significant differences in the primary outcomes evaluated: number of blood cultures collected to document a negative blood culture, time to SAB clearance, time to CVC placement, and duration of anti–*S aureus* IV therapy. However, we did observe signals that point to potential patient harm associated with severe restriction of blood cultures: higher 30-day hospital readmission rate, longer time to diagnosis for patients whose initial blood culture set was not positive, and the loss of early documentation of SAB clearance. The relatively low sample size (n = 36 in the intervention, n = 44 in the postintervention period) may have minimized any potential impact due to type II errors. Additionally, because the population studied was a convenience group (ie, those who presented to our hospital during the blood culture shortage), there is an imbalance in acuity between groups, which certainly affected outcomes to some degree.

It is impossible to fully assess the “missed opportunities” for diagnosis of SAB that resulted during our blood culture bottle shortage. Standard of care in adults is to collect 2–3 sets of blood cultures, each consisting of 20 mL of blood divided into 1 blood culture bottle that supports aerobic and 1 that supports anaerobic growth [17–20]. Across all 3 study periods, 96.3% of SAB cases were diagnosed on the first blood culture set collected (data not shown). It is important to note that >90% of SAB is detected in the first blood culture set performed [21]. In contrast, recovery of the Enterobacterales, *Pseudomonas aeruginosa*, and *Candida* is optimized by increasing the volume of blood per culture and the number of culture sets [20, 21]. As such, the missed opportunities during the blood culture shortage may have been more severe for other etiologies of bacteremia. Nonetheless, for the limited number of patients with delayed diagnosis of SAB, the time to diagnosis was substantially longer during the intervention period: 40.3 hours from initial culture collected in the intervention arm versus 10–18 hours in the pre-/postintervention arms. Importantly, all patients were treated empirically with vancomycin, which may be why outcomes did not differ between study periods. Nonetheless, the longer time to SAB diagnosis in the intervention period hints at the combined negative impact of restricting blood cultures to a single set and restricting repeat cultures to once every 48 hours. In other words, the rare case of SAB not detected on initial culture could be detected with repeat cultures, ordered in response to the patient's continued fever and/or signs of sepsis, provided the patient is not on active antimicrobial therapy.

On the other end of the diagnostic spectrum, while the number of cultures and days to document SAB clearance did not significantly differ across the study periods, we observed that early documentation of clearance (within 24 hours), which was available for 9% of patients in the preintervention cohort, was not observed for any patients in the intervention or postintervention cohorts (Figure 2, Table 2). Early documentation of clearance of bacteremia provides the opportunity to shorten time until CVC placement, reduce days of therapy, and potentially lead to earlier hospital discharge. Delays in discharge may prevent newly admitted patients from being placed in beds, lead to ED overcrowding and patient dissatisfaction, and increase risk of adverse events [22].

Finally, it is important to note that SAB has mandatory ID consultation at our primary teaching hospital, where approximately 60% of patients with SAB were treated (Table 1). The ID physicians and trainees communicate regularly with the microbiology laboratory and were able to request follow-up cultures when SAB was recognized and requested cultures be allowed within the 48-hour restriction period for 6 patients (16.7%) during the intervention period. During the shortage, roughly 3–4 calls per day were received by the microbiology attending for review and potential approval of exceptions to the blood culture shortage; most cases were requests for additional cultures to confirm a suspected contaminated culture. In addition, ED physicians could bypass the restrictions to order 2 initial sets of cultures for patients they deemed clinically to be septic; this occurred for 3 patients with SAB.

In summary, we showed no clear evidence of patient harm of restricting blood cultures for patients with SAB. Nonetheless, signals that did not achieve statistical significance, including increased readmission rates, delays in diagnosis, and reduced opportunity to document SAB clearance within 24 hours, demonstrate that these restrictions were not without potential impact on the individual patient level. Following the intervention period, we returned to a strong recommendation for the collection of 2–3 blood culture sets for initial diagnosis. We maintained general guidance on when blood cultures are indicated, as well as guidance to consider delaying repeat cultures to ≥48 hours from the initial culture. For this latter guidance, we highlight several instances where repeat cultures may be indicated, including for patients with SAB or highly resistant gram-negative bacteria, following an initial contaminated culture, or if the patient displays new onset of sepsis. In practice, we observed that repeat cultures occurred within 48 hours for 45% of patients in the postintervention cohort, as compared to 12% in the preintervention cohort. Further study will be required to determine the long-term consequences of limiting repeat blood cultures until after 48 hours. Roughly 85% of blood cultures collected at our institution are negative and may be unnecessary. Blood cultures are associated with the risk of contamination and overtreatment, and careful stewardship may be an effective strategy to minimize this risk [13].

## Supplementary Material

ofaf546_Supplementary_Data

## Supplementary Data

Supplementary materials are available at *Open Forum Infectious Diseases* online. Consisting of data provided by the authors to benefit the reader, the posted materials are not copyedited and are the sole responsibility of the authors, so questions or comments should be addressed to the corresponding author.

## References

[1] Laupland KB, Lyytikainen O, Sogaard M, et al The changing epidemiology of *Staphylococcus aureus* bloodstream infection: a multinational population-based surveillance study. Clin Microbiol Infect 2013; 19:465–71. 22616816 10.1111/j.1469-0691.2012.03903.x

[2] Nambiar K, Seifert H, Rieg S, et al Survival following *Staphylococcus aureus* bloodstream infection: a prospective multinational cohort study assessing the impact of place of care. J Infect 2018; 77:516–25. 30179645 10.1016/j.jinf.2018.08.015

[3] Thwaites GE, Scarborough M, Szubert A, et al Adjunctive rifampicin for *Staphylococcus aureus* bacteraemia (ARREST): a multicentre, randomised, double-blind, placebo-controlled trial. Lancet 2018; 391:668–78. 29249276 10.1016/S0140-6736(17)32456-X PMC5820409

[4] Lopez-Cortes LE, Del Toro MD, Galvez-Acebal J, et al Impact of an evidence-based bundle intervention in the quality-of-care management and outcome of *Staphylococcus aureus* bacteremia. Clin Infect Dis 2013; 57:1225–33. 23929889 10.1093/cid/cit499

[5] Vogel M, Schmitz RP, Hagel S, et al Infectious disease consultation for *Staphylococcus aureus* bacteremia—a systematic review and meta-analysis. J Infect 2016; 72:19–28. 26453841 10.1016/j.jinf.2015.09.037

[6] Miller JM, Binnicker MJ, Campbell S, et al Guide to utilization of the microbiology laboratory for diagnosis of infectious diseases: 2024 update by the Infectious Diseases Society of America (IDSA) and the American Society for Microbiology (ASM) [manuscript published online ahead of print 5 March 2024]. Clin Infect Dis 2024. doi:10.1093/cid/ciae104 38442248

[7] Kuehl R, Morata L, Boeing C, et al Defining persistent *Staphylococcus aureus* bacteraemia: secondary analysis of a prospective cohort study. Lancet Infect Dis 2020; 20:1409–17. 32763194 10.1016/S1473-3099(20)30447-3

[8] Tande AJ, Palraj BR, Osmon DR, et al Clinical presentation, risk factors, and outcomes of hematogenous prosthetic joint infection in patients with *Staphylococcus aureus* bacteremia. Am J Med 2016; 129:221 e11–20. 10.1016/j.amjmed.2015.09.006 26453989

[9] Humphries RM, Wright PW, Banerjee R, et al Rapid implementation of blood culture stewardship: institutional response to an acute national blood culture bottle shortage. Clin Infect Dis 2025; 80:472–4. 39136555 10.1093/cid/ciae402 PMC11848254

[10] Fabre V, Sharara SL, Salinas AB, Carroll KC, Desai S, Cosgrove SE. Does this patient need blood cultures? A scoping review of indications for blood cultures in adult nonneutropenic inpatients. Clin Infect Dis 2020; 71:1339–47. 31942949 10.1093/cid/ciaa039

[11] Liu C, Bayer A, Cosgrove SE, et al Clinical practice guidelines by the Infectious Diseases Society of America for the treatment of methicillin-resistant *Staphylococcus aureus* infections in adults and children. Clin Infect Dis 2011; 52:e18–55. 21208910 10.1093/cid/ciq146

[12] Baddour LM, Wilson WR, Bayer AS, et al Infective endocarditis in adults: diagnosis, antimicrobial therapy, and management of complications: a scientific statement for healthcare professionals from the American Heart Association. Circulation 2015; 132:1435–86. 26373316 10.1161/CIR.0000000000000296

[13] Fabre V, Klein E, Salinas AB, et al A diagnostic stewardship intervention to improve blood culture use among adult nonneutropenic inpatients: the DISTRIBUTE study. J Clin Microbiol 2020; 58:e01053-20. 32759354 10.1128/JCM.01053-20 PMC7512168

[14] Abraham L, Bamberger DM. *Staphylococcus aureus* bacteremia: contemporary management. Mo Med 2020; 117:341–5. 32848271 PMC7431060

[15] Henderson H, Luterbach CL, Cober E, et al The Pitt bacteremia score predicts mortality in nonbacteremic infections. Clin Infect Dis 2020; 70:1826–33. 31219148 10.1093/cid/ciz528 PMC7156778

[16] Choi SH, Dagher M, Ruffin F, et al Risk factors for recurrent *Staphylococcus aureus* bacteremia. Clin Infect Dis 2021; 72:1891–9. 32564065 10.1093/cid/ciaa801 PMC8315037

[17] Weinstein MP, Murphy JR, Reller LB, Lichtenstein KA. The clinical significance of positive blood cultures: a comprehensive analysis of 500 episodes of bacteremia and fungemia in adults. II. Clinical observations, with special reference to factors influencing prognosis. Rev Infect Dis 1983; 5:54–70. 6828812 10.1093/clinids/5.1.54

[18] Washington J . Subject review: blood culture, principles and techniques. Mayo Clin Proc 1975; 50:91–8. 1090789

[19] Cockerill FR 3rd, Wilson JW, Vetter EA, et al Optimal testing parameters for blood cultures. Clin Infect Dis 2004; 38:1724–30. 15227618 10.1086/421087

[20] Weinstein MP, Mirrett S, Wilson ML, Reimer LG, Reller LB. Controlled evaluation of 5 versus 10 milliliters of blood cultured in aerobic BacT/Alert blood culture bottles. J Clin Microbiol 1994; 32:2103–6. 7814532 10.1128/jcm.32.9.2103-2106.1994 PMC263950

[21] Lee A, Mirrett S, Reller LB, Weinstein MP. Detection of bloodstream infections in adults: how many blood cultures are needed? J Clin Microbiol 2007; 45:3546–8. 17881544 10.1128/JCM.01555-07 PMC2168497

[22] Patel H, Fang MC, Mourad M, et al Hospitalist and internal medicine leaders' perspectives of early discharge challenges at academic medical centers. J Hosp Med 2018; 13:388–91. 29240850 10.12788/jhm.2885

