# Supplementary material for: Association Between Blood Culture Bottle Shortage and Ordering Restrictions and Clinical Outcomes for Patients With Staphylococcus aureus Bacteremia
Source: Open Forum Infect Dis. 2025 Sep 15;12(9):ofaf546. doi: 10.1093/ofid/ofaf546 (PMC12448447; doi:10.1093/ofid/ofaf546)
Supplement: ofaf546_Supplementary_Data [file ofaf546_supplementary_data.pdf]

Supplemental Figure 1. Educational flyer used for blood culture indications at VUMC during the shortage

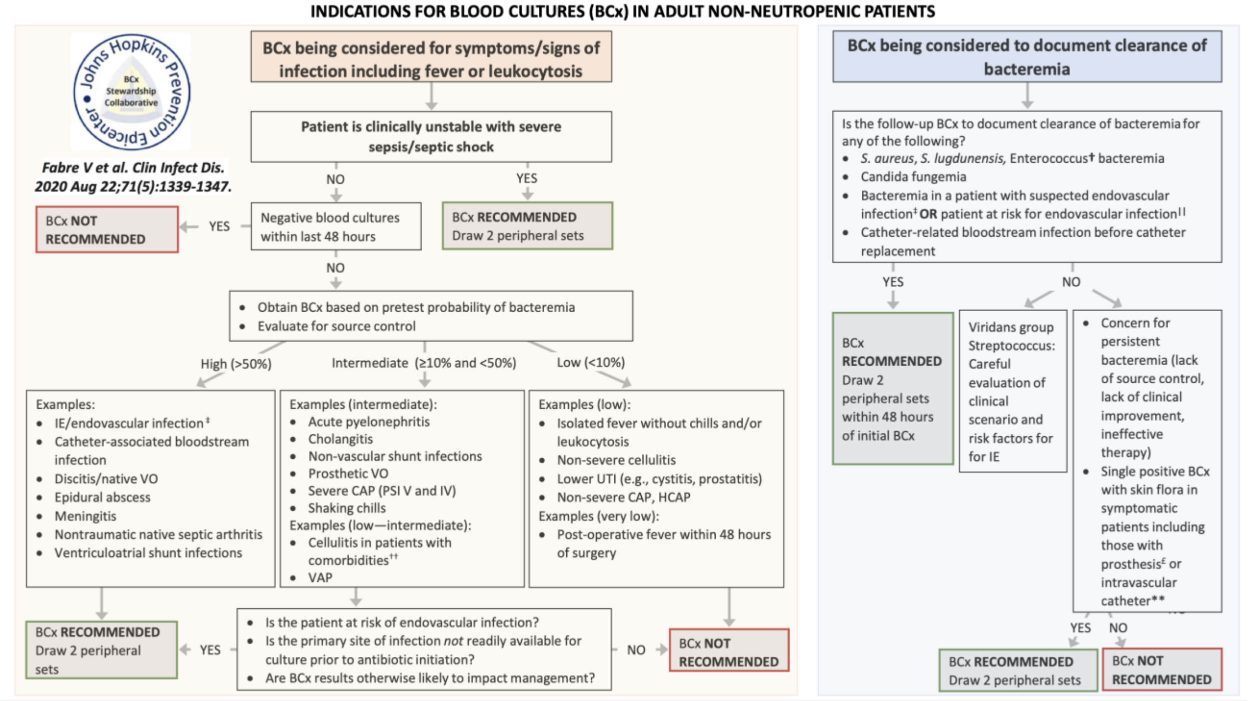

Supplemental Figure 2. Education embedded in the Hospital Electronic Medical Record order panel for blood cultures. Indications for high, intermediate and low probability of bacteremia were as listed in Figure S1.

Temporary recommendations due to national blood culture media shortage

Testing for symptoms/signs of infection incl. fever or leukocytosis:
-Patient is clinically unstable/signs of sepsis ▸ Blood cultures indicated
-Negative blood cultures in past 48 hours ▸ Repeat blood cultures NOT indicated
-Decide to obtain blood cultures based on pre-test probability of bacteremia:
- ⓘ High (>50%, hover for examples) ▸ Blood cultures indicated
- ⓘ Intermediate (between 10-50%, hover for examples) ▸ ⓘ Blood cultures may be indicated (hover for details)
- ⓘ Low (<10%, hover for examples) ▸ Blood cultures NOT indicated

Testing to document clearance of bacteremia:
-Blood cultures indicated for the following:
- The patient had bacteremia due to Staph aureus or lugdenensis, Enterococcus, or Candida species
- ⓘ There is concern for persistent bacteremia/lack of source control (hover for details)
- The patient had a common skin flora grow in initial blood culture and has a prosthesis or vascular catheter

Collection of more than 1 set in 48 hours is restricted
Allow existing blood culture specimens time to turn positive before ordering another set.
NO ORDERS FOR BLOOD CULTURES IN THE ABSENCE OF CLINICAL SIGNS/SYMPTOMS (e.g. As a routine preoperative test).
NO STANDING ORDERS FOR BLOOD CULTURES
NO ORDERS FOR DAILY COLLECTION OF BLOOD CULTURES

Set = 1 aerobic and 1 anaerobic culture bottle
